# Severity and Vaccine Effectiveness in Patients With the Omicron Variant of COVID-19 in Suzhou: A Retrospective Single-Center Study

**DOI:** 10.7759/cureus.41200

**Published:** 2023-06-30

**Authors:** Yanmei Cao, Jianping Zhang, Yiming Zhao, Fen Hui, Zhijie Hu, Xinhua Shen

**Affiliations:** 1 Department of Occupational Medicine, The Affiliated Infectious Diseases Hospital of Soochow University, The Fifth People’s Hospital of Suzhou, Suzhou, CHN; 2 Department of Tuberculosis Medicine, The Affiliated Infectious Diseases Hospital of Soochow University, The Fifth People’s Hospital of Suzhou, Suzhou, CHN; 3 Department of Medical Section, The Affiliated Infectious Diseases Hospital of Soochow University, The Fifth People’s Hospital of Suzhou, Suzhou, CHN

**Keywords:** clinical characteristics, vaccine, omicron, sars-cov-2, coronavirus disease 2019

## Abstract

Background

The Omicron variant of the coronavirus disease 2019 (COVID-19) virus has spread rapidly worldwide, even in areas with high vaccination rates. Consequently, it has further exacerbated the current global pandemic. In this study, we aimed to characterize the clinical severity of patients with the COVID-19 variant Omicron and analyze vaccine effectiveness in predicting clinical severity.

Methodology

A total of 142 patients who contracted the COVID-19 virus in the Omicron era were retrospectively studied, and differences in their clinical severity were analyzed. They were stratified as follows: unvaccinated vs. vaccinated, unvaccinated vs. one to two vaccine doses vs. three vaccine doses, and cycle threshold (CT) values ≤ 28 vs. CT > 28.

Results

Of the 142 patients, 27 were asymptomatic, 83 had mild disease, and 32 had moderate disease. The median age was 32 years for asymptomatic patients vs. 31 years for those with mild disease vs. 59 years for those with moderate disease (P＜0.05), and the direct medical hospitalization costs were ¥4901 for asymptomatic patients vs. ¥5259 for those with mild disease vs. ¥8378 for those with moderate disease (P＜0.05). Of the 142 patients, 112 (78.8%) were vaccinated, 11 (7.7%) had one vaccine dose, 63 (44.4%) had two vaccine doses, and 38 (26.7%) received three vaccine doses. The median direct medical cost in the vaccinated group was significantly lower than that in the unvaccinated group (¥5470.5 vs. ¥7535.5, P＜0.05). For ORF1ab and N genes, hospital stay length and direct medical cost significantly decreased in the group with CT values > 28 compared with those in the group with CT values ≤ 28 (P＜0.05). Multiple regression analysis showed that being ≥ 60 years old could be a predictor of moderate disease severity in patients, and three vaccine doses could be effective against moderate COVID-19.

Conclusion

Mild infection is the main clinical manifestation of the Omicron variant. Vaccination can significantly decrease direct Omicron-associated medical costs. Although vaccination cannot provide protection against severe disease caused by this variant, three vaccine doses are highly effective in preventing moderate COVID-19.

## Introduction

Since coronavirus disease 2019 (COVID-19) was first reported in Wuhan, China, in December 2019, the world has witnessed multiple waves of this global pandemic. As of April 12, 2023, 762,791,152 confirmed COVID-19 cases, including 6,897,025 deaths reported to the World Health Organization (WHO), have been documented [1]. In China, from January 3, 2020, to April 12, 2023, 99,239,252 cases of COVID-19 were confirmed and 120,905 deaths were documented [2]. Furthermore, this disease has severely affected the global economy and restricted travel, thereby affecting millions of people and putting a burden on medical staff, who have consequently suffered mentally, physically, and emotionally [3].

COVID-19 waves are caused by the original severe acute respiratory syndrome coronavirus 2 (SARS-CoV-2), which has mutated into several variants, including Alpha, Beta, Gamma, Delta, and Omicron [4]. Among them, Omicron is the most recently emerged variant of concern because it has spread quickly worldwide since it was first identified in Botswana and South Africa in early November 2021 [5]. On January 6, 2022, this variant reached Hong Kong, China, causing the fifth wave of COVID-19 cases [6]. Since then, Omicron variants such as BA.2, BA.2.2, BA.5, and BA.5.2 have spread to several cities in China [7].

When this study was conducted, a 100% curative regimen was not available, but there were treatments with some modest efficacy. Since the earliest stages of the COVID-19 pandemic, global efforts have been devoted to administering the COVID-19 vaccine to control this disease [8]. Vaccines are considered the most cost-effective way of controlling diseases, including COVID-19. In China, as of July 22, 2022, 3,416,727,000 vaccine doses had been administered, and the coverage rate of one dose, full dose, and booster vaccination of the total population was 92.1%, 89.7%, and 71.7%, respectively [9].

Because of the effective implementation of the policy of "zero tolerance for local transmission" in China, only two distinct local small-scale COVID-19 epidemic events occurred in Suzhou, where the Omicron variant caused a second breakout from February 13, 2022, to March 2, 2022. This study aimed to characterize the clinical severity of the COVID-19 variant Omicron in Suzhou and analyze vaccine effectiveness in predicting clinical severity.

## Materials and methods

This study was approved by the Medical Ethics Committee of the Affiliated Infectious Diseases Hospital of Soochow University, Suzhou, China (2022/009); thus, the need for informed consent was waived on the basis of the observational and non-interventional nature of the study. Patient information remained anonymous; no sensitive personal data were used. This manuscript was compliant with the Strengthening the Reporting of Observational Studies in Epidemiology (STROBE) guidelines for cross-sectional studies.

Study design and participants

This retrospective single-center study was conducted from February 13, 2022, to March 2, 2022. A total of 142 consecutive patients were admitted and treated in the Fifth People’s Hospital of Suzhou, and all of them were diagnosed with COVID-19 Omicron variant BA.2.2. The clinical criteria for diagnosis and discharge were in accordance with the standards for "Diagnosis and Treatment Scheme of New Coronavirus-Infected Pneumonia" (trial version 9) [10]. All patients were diagnosed via reverse transcription polymerase chain reaction (RT-PCR), and the cycle threshold (CT) values of two genes, ORF1ab and N, were determined. The following discharge criteria were applied: two consecutive novel coronavirus tests for ORF1ab and N CT values of ≥ 35 and at least a 24-hour interval between the two samples [10]. Multiple rounds of nucleic acid tests were conducted by the government in the whole city, and all patient records with COVID-19 were included.

Data collection

The clinical data of the patients were acquired using the hospitalization management system. Relevant data were extracted and collected by three doctors after undergoing training on the data abstraction form. The following data were extracted: age, sex, vaccination status, hospital stay length, direct medical cost, comorbidities, severity classification (Appendix A), and CT values.

Coronavirus detection

Oropharyngeal swabs were collected by a trained nurse, and specimens were examined using a novel coronavirus (2019-nCoV) nucleic acid detection kit (PCR-fluorescence probing; BioGerm Medical Biotechnology Co., Ltd., Shanghai, China; BioGerm kit), which targets ORF1ab (encoding 16 nonstructural proteins) and N (encoding the nucleocapsid protein) genes of SARS‐CoV‐2. Specimens with CT values > 40 were negative for COVID-19.

Statistical analysis

Mean and standard deviation, or median with interquartile range (IQR), were reported for continuous variables. Normally distributed continuous variables were analyzed via the Student’s t-test or one-way Analysis of variance (ANOVA). Non-normally distributed continuous variables were compared via a Kruskal-Wallis test. Categorical variables were reported as numbers and percentages and then analyzed via a chi-squared test or Fisher’s exact test. Predictors associated with moderate cases of COVID-19 were identified through multivariate analysis and controlled in a screening model through multiple logistic regression. The data were statistically analyzed using Statistical Analysis System (SAS) version 9.3 (SAS Institute Inc., Cary, North Carolina, USA) and considered statistically significant at P<0.05.

## Results

Clinical characteristics

The records of 142 patients who contracted the COVID-19 virus in the Omicron era were included in the study. The median age of the patients was 34.5 years (IQR, 25-47 years), and 74 (52.1%) subjects were male. The patients were divided into three groups to analyze their clinical characteristics: asymptomatic patients (n=27), patients with mild disease (n=83), and patients with moderate disease (n=32). The groups did not significantly differ in sex, vaccine, length of hospital stay, diabetes, or CT values. Hypertension was highly prevalent in the moderate group (28.1% of the subjects) and significantly more frequent in the moderate group than in the asymptomatic (3.7% of the subjects) and mild disease (7.2% of the subjects) groups (P=0.003). The median direct medical cost of the moderate group was ¥8387, which was significantly higher than that of the asymptomatic (¥4901) and mild (¥5259) groups (P<0.001). The basic demographic and clinical characteristics of the patients are shown in Table 1.

**Table 1:** Basic demographics and clinical characteristics of patients CT: cycle threshold; ¥: Chinese Yuan

Variable	All (N=142)	Asymptomatic (N=27)	Mild (N=83)	Moderate (N=32)	P-value
Age, years	34.50 (25.00,47.00)	32.00 (20.00,39.00)	31.00 (24.00,41.00)	59.00 (34.00,68.00)	<0.001
Age group, years					<0.001
< 18	21 (14.79)	6 (22.22)	67 (80.72)	18 (56.25)	
18-59	105 (73.94)	20 (74.07)	3 ( 3.61)	12 (37.50)	
≥ 60	16 (11.27)	1 ( 3.70)	13 (15.66)	2 ( 6.25)	
Sex, male, n(%)	74 (52.11)	16 (59.26)	44 (53.01)	14 (43.75)	0.478
Vaccine, n(%)	112 (78.87)	24 (88.89)	66 (79.52)	22 (68.75)	0.164
Vaccine group, n(%)					0.049
Unvaccinated	30(21.13)	10 (31.25)	17 (20.48)	3 (11.11)	
One to two vaccine doses	74(52.11)	19 (59.38)	38 (45.78)	17 (62.96)	
Three vaccine doses	38(26.76)	3 ( 9.38)	28 (33.73)	7 (25.93)	
Hospital stay length, days	15.00 (12.00,18.00)	14.00 (12.00,18.00)	15.00 (12.00,17.00)	16.00 (12.00,19.00)	0.368
Direct medical cost, ¥	5638.50 (4514.00,8010.00)	4901.00 (4049.00,6062.00)	5259.00 (4357.00,7217.00)	8378.00 (6696.00,12585.50)	<0.001
Comorbidities, n(%)					
Hypertension	16 (11.27)	1 ( 3.70)	6 ( 7.23)	9 (28.13)	0.003
Diabetes	4 ( 2.82)	0 ( 0.00)	2 ( 2.41)	2 ( 6.25)	0.372
Others	5 ( 3.52)	2 ( 7.40)	0 ( 0.00)	3 ( 9.37)	0.014
CT value ORF1ab	24.56 ± 4.34	24.76 (20.52,27.65)	24.30 (21.12,27.24)	22.82 (20.75,26.78)	0.804
CT value N	25.10 ± 4.10	25.62 (21.93,28.65)	24.69 (21.78,28.35)	23.80 (21.67,26.82)	0.714

Vaccine effectiveness

Of the 142 patients, 112 (78.8%) were vaccinated, 11 (7.7%) had one vaccine dose, 63 (44.4%) had two vaccine doses, and 38 (26.7%) had received three vaccine doses. Patients with the COVID-19 Omicron variant were divided into two groups (unvaccinated and vaccinated groups) and further divided into three groups (unvaccinated group vs. groups with one to two vaccine doses vs. group with three vaccine doses) to analyze vaccine effectiveness. The median direct medical cost of the vaccinated group was significantly lower than that of the unvaccinated group (P=0.008). When the patients were divided into three groups, the direct medical cost significantly decreased in the group that received three vaccine doses compared with those in the group that received one to two vaccine doses and the unvaccinated group (P<0.05). The compared demographic and clinical characteristics of vaccination status are shown in Table 2.

**Table 2:** Comparison between the demographics of the study group and the clinical characteristics of vaccination status CT: cycle threshold; ¥: Chinese Yuan

Variable	Unvaccinated (N=30)	Vaccinated (N=112)	P-value		Unvaccinated (N=30)	One to two vaccine doses (N=74)	Three vaccine doses (N=38)	P-value
Age, years	38.00 (23.00,68.00)	34.00 (25.00,43.50)	0.308		38.00 (23.00,68.00)	32.00 (22.00,50.00)	36.50 (28.00,41.00)	0.362
Age group, years			0.001					＜0.001
< 18	5 (16.67)	16 (14.29)			5 (16.67)	16 (21.62)	0 ( 0.00)	
18-59	16 (53.33)	89 (79.46)			16 (53.33)	53 (71.62)	36 (94.74)	
≥ 60	9 (30.00)	7 ( 6.25)			9 (30.00)	5 ( 6.76)	2 ( 5.26)	
Sex, male, n(%)	17 (56.67)	57 (50.89)	0.574		17 (56.67)	34 (45.95)	23 (60.53)	0.293
Direct medical cost, ¥	7535.50 (5068.00,12512.00)	5470.50 (4443.00,7377.00)	0.008		7535.50 (5068.00,12512.00)	5532.00 (4414.00,7184.00)	5344.50 (4543.00,7548.00)	0.030
Hospital stay length, days	16.00 (14.00,19.00)	14.00 (12.00,17.00)	0.060		16.00 (14.00,19.00)	14.00 (12.00,17.00)	14.50 (12.00,17.00)	0.168
Severity classification, n(%)			0.164					0.049
Asymptomatic	3 (10.00)	24 (21.43)			3 (10.00)	17 (22.97)	7 (18.42)	
Mild	17 (56.67)	66 (58.93)			17 (56.67)	38 (51.35)	28 (73.68)	
Moderate	10 (33.33)	22 (19.64)			10 (33.33)	19 (25.68)	3 ( 7.89)	
Comorbidities, n(%)								
Hypertension	6 (20.00)	10 ( 8.93)	0.106		6 (20.00)	7 ( 9.46)	35 (92.11)	0.227
Diabetes	2 ( 6.67)	2 ( 1.79)	0.196		2 ( 6.67)	1 ( 1.35)	2 ( 6.67)	0.326
Others	3 (10.00)	2 ( 1.78)	0.063		3 (10.00)	1 ( 1.35)	1 ( 2.63)	0.058
CT value ORF1ab	22.98 (20.08,27.59)	24.29 (21.41,27.28)	0.236		22.98 (20.08,27.59)	24.47 (21.56,27.54)	24.17 (21.16,26.28)	0.404
CT value N	22.49 (21.28,29.04)	24.81 (22.06,27.85)	0.357		22.49 (21.28,29.04)	24.81 (22.19,28.52)	24.89 (21.93,26.75)	0.580

RT-PCR CT values

The subjects were stratified into two groups according to the diagnostic CT values detected from the first swab as a basis for diagnosing a SARS-CoV-2 infection to further analyze the relationship between the CT values and clinical characteristics of patients with COVID-19. They were grouped on the basis of CT values ≤ 28, and CT values > 28 [11]. Among the frequency of all subjects with CT values > 28, 26 (18.3%) patients had the ORF1ab gene, and 36 (25.3%) patients had N. For the ORF1ab group, the median age with CT values > 28 was significantly lower than that of the group with CT values ≤ 28 (P<0.05). The hospital stay length and direct medical cost significantly decreased in the group with CT values > 28 compared with those in the group with CT values ≤ 28 (P<0.05). For N group, the median age of the group with CT values > 28 tended to be lower (P=0.067). The hospital stay length and direct medical cost significantly decreased in the group with CT values > 28 compared with those in the group with CT values ≤ 28 (P<0.05). However, the groups did not significantly differ in vaccine history or vaccine groups. The comparisons of demographic and clinical characteristics between CT value groups are shown in Table 3.

**Table 3:** Comparison of the demographics of the study group and clinical characteristics between the CT value groups

Variable	ORF1ab				N		
	≤28 (N=116)	＞28 (N=26)	P value		≤28 (N=106)	＞28 (N=36)	P-value
Age, years	36.00 (26.50,53.50)	29.50 (8.00,36.00)	0.010		36.00 (26.00,54.00)	30.50 (9.00,43.00)	0.067
Age group, years			0.003				0.013
< 18	12 (10.34)	9 (34.62)			11 (10.38)	10 (27.78)	
18-59	88 (75.86)	17 (65.38)			80 (75.47)	25 (69.44)	
≥ 60	16 (13.79)	0 ( 0.00)			15 (14.15)	1 ( 2.78)	
Sex, male, n(%)	59 (50.86)	15 (57.69)	0.529		53 (50.00)	21 (58.33)	0.387
Vaccine, n(%)	92 (79.31)	20 (76.92)	0.788		84 (79.25)	28 (77.78)	0.852
Vaccine group, n(%)			0.631				0.509
Unvaccinated	24 (20.69)	6 (23.08)			22 (20.75)	8 (22.22)	
One to two vaccine doses	59 (50.86)	15 (57.69)			53 (50.00)	21 (58.33)	
Three vaccine doses	33 (28.45)	5 (19.23)			31 (29.25)	7 (19.44)	
Hospital stay length, days	15.00 (13.00,18.00)	12.00 (11.00,15.00)	0.002		15.50 (13.00,19.00)	13.00 (11.00,15.00)	0.001
Direct medical cost, ¥	5758.00 (4777.50,8309.50)	4970.50 (3900.00,6630.00)	0.017		5781.50 (4826.00,8395.00)	4855.00 (3898.00,6615.00)	0.003
Severity classification, n(%)			0.871				0.170
Asymptomatic	23 (19.83)	4 (15.38)			17 (16.04)	10 (27.78)	
Mild	67 (57.76)	16 (61.54)			62 (58.49)	21 (58.33)	
Moderate	26 (22.41)	6 (23.08)			27 (25.47)	5 (13.89)	
Comorbidities, n(%)							
Hypertension	12 (10.34)	4 (15.38)	0.494		11 (10.38)	5 (13.89)	0.552
Diabetes	2 ( 1.72)	2 ( 7.69)	0.153		2 ( 1.89)	2 ( 5.56)	0.266
Others	4 ( 3.44)	1 ( 3.84)	1.000		4 ( 3.77)	1 ( 2.77)	1.000

Predictors of moderate severity among patients with the COVID-19 variant Omicron

The subjects aged ≥ 60 years had a 25.1-fold higher risk of moderate COVID-19 than those aged < 18 years (95% confidence interval (CI): 3.68, 171.58); furthermore, the subjects aged ≥ 60 years had a 10.3-fold higher risk of moderate COVID-19 than those aged 18-59 years (95% CI: 2.81, 38.35). In this analysis, 20% (95% CI: 0.05, 0.83) of the subjects who received three vaccine doses were protected against moderate COVID-19. When controlled with other factors in the screening model, these results were consistent with the odds ratios (ORs) of 36.5 (95% CI: 5.53, 240.94), 12.8 (95% CI: 3.55, 46.46), and 0.20 (95% CI: 0.05, 0.80). The predictors of moderate severity among patients with COVID-19 are shown in Table 4.

**Table 4:** Predictors of moderate severity among patients with COVID-19 Model 1: Enforce potential risk variables as independent variables. Model 2: On the basis of model 1, independent variables were screened and statistically significant variables were retained. OR: odds ratio; CI: confidence interval

		Model 1			Model 2	
Variable	Variable	OR (95% CI)	P-value		OR (95% CI)	P-value
Age						
	≥60 years vs. <18 years	25.15 (3.68, 171.85)	＜0.001		36.53 (5.53, 240.94)	＜0.001
	≥60 years vs. 18-59 years	10.38 (2.81, 38.35)	＜0.001		12.85 (3.55, 46.46)	＜0.001
	18-59 years vs. <18 years	2.42 (0.50, 11.69)	0.187			
Sex	Male vs. Female	0.90 (0.35, 2.25)	0.824			
Vaccine	Three doses vs. ≤ two doses	0.20 (0.05, 0.83)	0.026		0.20 (0.05, 0.80)	0.022
Hypertension	No vs. Yes	0.31 (0.08, 1.11)	0.291			
Diabetes	No vs. Yes	1.69 (0.13, 21.84)	0.686			

## Discussion

This study describes the distribution of clinical severity among patients with the COVID-19 variant Omicron in Suzhou. Among them, 19.0% were asymptomatic, 58.5% had a mild infection, and 22.5% had a moderate infection. We also found that patients ≥ 60 years had a higher risk of having moderate COVID-19. In contrast to the records during the first wave of COVID-19 in 2020, the records in 2022 showed that no patients had a severe or critical infection. In the Omicron wave vs. previous waves, the patients had a median age of 34.5 years vs. 44.5 years. The lengths of hospital stay were 15 and 21 days, and direct medical costs were ¥5638 vs. ¥17850, respectively.

COVID-19 remains a global pandemic. Worldwide, Omicron has been identified in 57 countries, and the number of confirmed Omicron variant cases has significantly increased [12]. Thus far, the Omicron variant is the most mutated among SARS‐CoV‐2 variants [13]. Nevertheless, its major clinical manifestation is a mild infection; for younger and middle-aged people, its infectivity is higher than that of previous variants [12]. The percentage of deaths and intensive care unit (ICU) admissions of patients infected with the Omicron variant is significantly lower than that of previous waves, and the hospital stay is significantly shorter [14]. Only one-third of the patients had COVID-19 pneumonia; of these patients, 72% had mild to moderate disease [14]. Many of the first reported cases of Omicron variant infection are mild, and symptoms are expected to be milder in vaccinated individuals [15]. In China, because of the effective implementation of the policy of "zero tolerance for local transmission," only small amputation-related outbreaks occurred. The two distinct local small-scale epidemic waves of SARS-CoV-2 in Suzhou were driven by different variants: the first event that occurred in February 2020 was associated with the original SARS-CoV-2, while the second wave that began in February 2022 was driven by the Omicron variant. Even if most infections are mild, a highly transmissible variant can result in sufficient cases to overwhelm health systems. Thus, the clinical severity of Omicron infection should be further elucidated by identifying and investigating additional cases [15].

The WHO claimed that the most efficient way to end the COVID-19 pandemic was through safe and effective vaccination [8]. In total, 70.3% of the world’s population has received at least one dose of the COVID-19 vaccine [16]. Currently, 13.47 billion doses have been administered globally, and 93,683 are now administered each day [16]. In this study, in the moderate group, as the vaccine dose increased, the percentage of patients decreased. Multivariate regression analysis showed that three vaccine doses were highly effective in preventing moderate COVID-19. Vaccines are highly effective against symptomatic diseases, severe diseases, and fatal outcomes caused by the original SARS-CoV-2 strain and the alpha variant [17-20]. Variants of concern are new genetic versions of the virus with increased transmissibility, a change in virulence or disease presentation, or a decrease in the effectiveness of mitigation measures, available vaccines, or treatments [21]. Previous studies suggested reduced vaccine effectiveness against infection and hospital admissions for Omicron compared with that against earlier variants [22-24]. Two or three messenger RNA (mRNA) vaccines can be effective in preventing COVID-19, i.e., 65% and 86% against the Omicron variant, respectively [21]. COVID-19 severity according to the WHO clinical progression scale is substantially lower for vaccinated cases than for unvaccinated cases in the Omicron variant group [21]. However, the vaccine’s protective effect against the Omicron variant is controversial. In patients who received two vaccine doses, vaccination almost had no protective effect against the symptomatic disease caused by the Omicron variant from 20 weeks to 24 weeks after the second dose [5]. Our findings support the maximum coverage of the third dose of vaccine in the population in Suzhou, but further studies should be performed to assess the protective effect against severe disease.

In this study, vaccination was not associated with CT values at the early stages of infection. An earlier cohort study showed that the CT values are higher for breakthrough infections than those for reinfections in unvaccinated individuals [25]. Another study has indicated that CT values in specimens from vaccinated individuals with breakthrough cases are similar to those from unvaccinated and partially vaccinated individuals or from those with unknown vaccination status [26]. Yet some studies suggest that vaccinated individuals who acquire infections may be less infectious than unvaccinated ones, as vaccine-primed immune responses may attenuate the natural history of infection by reducing viral replication and accelerating viral clearance, leading to a lower viral load and higher CT values [27, 28]. Our study indicated that high CT values were associated with a reduction in the length of hospital stay and cost; therefore, it could potentially reduce healthcare system resource needs, which are critically important in current and future pandemic situations [29].

Limitations

The study has several limitations, and the findings should be carefully interpreted. First, the study was conducted in a single center, and the sample size was limited; therefore, a large-scale study should be performed for further implementation. Second, our comparison group included unvaccinated patients, who accounted for a small percentage of the total subjects. Third, only vaccines were evaluated in this study; however, the effects of different types of vaccines were not examined. Fourth, the duration of effectiveness of the COVID-19 vaccine after the patients received it was not assessed in this study. Fifth, CT values did not differ in terms of vaccine or severity, which is subjected to oropharyngeal swab sampling and the procedure tolerance of the individual tested. In future studies, the duration of protection, protection against future variants, and the effect of vaccines on infectivity should be determined.

## Conclusions

The main clinical manifestation of patients with COVID-19 variant Omicron was mild infection; patients older than 60 years had a higher risk of having moderate COVID-19. Three vaccine doses effectively prevent moderate COVID-19 caused by the Omicron variant. Although breakthrough infections have been observed globally, the public health benefits of vaccination should be continuously evaluated. Therefore, these findings emphasize the urgency of scaling up vaccination globally to control the extent of the pandemic.

## Appendices

Appendix A 

**Table 5:** Severity classification of COVID-19 COVID-19: coronavirus disease 2019 According to the “Diagnosis and Treatment Scheme of New Coronavirus Infected Pneumonia” (trial version 9) by the National Health Commission

Type	Clinical feature
Asymptomatic	No clinical symptoms or abnormal radiological findings
Mild	The clinical symptoms are mild, with no abnormal radiological findings
Moderate	Fever, cough, and other symptoms are presented with viral pneumonia on chest computed tomography
Severe	One of the following conditions is met
	(1) Respiratory distress, respiratory rate ≥ 30 per min
	(2) Oxygen saturation on room air at rest ≤ 93%
	(3) Partial pressure of oxygen in arterial blood/fraction of inspired oxygen ≤ 300 mmHg
Critical	One of the following conditions has to be met
	(1) Respiratory failure occurs and mechanical ventilation is required
	(2) Shock occurs
	(3) Patients with other organ dysfunction needing intensive care unit monitoring treatment
